# Factors associated with self-reported exposure to chemical substances at work in Brazil: results from the National Health Survey, 2013

**DOI:** 10.11606/s1518-8787.2020054001461

**Published:** 2020-08-28

**Authors:** Ada Ávila Assunção, Mery Natali Silva Abreu, Priscila Sílvia Nunes Souza

**Affiliations:** I Universidade Federal de Minas Gerais Faculdade de Medicina Departamento de Medicina Preventiva e Social Belo HorizonteMG Brasil Universidade Federal de Minas Gerais. Faculdade de Medicina. Departamento de Medicina Preventiva e Social. Belo Horizonte, MG, Brasil; II Universidade Federal de Minas Gerais Escola de Enfermagem Departamento de Gestão em Saúde Belo HorizonteMG Brasil Universidade Federal de Minas Gerais. Escola de Enfermagem. Departamento de Gestão em Saúde. Belo Horizonte, MG, Brasil; III Universidade Federal de Minas Gerais Escola de Enfermagem Belo HorizonteMG Brasil Universidade Federal de Minas Gerais. Escola de Enfermagem. Belo Horizonte, MG, Brasil

**Keywords:** Occupational exposure, Chemical Compounds, Working conditions, Occupational Health, Health Surveys, National Survey on Health

## Abstract

**OBJECTIVE:**

To describe the prevalence of self-reported exposure to chemical substances at work and its associated factors in a sample of Brazilian adults that participated in the National Health Survey, conducted between 2013 and 2014.

**METHODS:**

Our sample consisted of adults aged 18 years or older that answered question E1 of module E: “In the week of July 21-27, 2013 (reference week), did you work as regular employee or intern for at least an hour in any activity paid with cash?” Sociodemographic data, situation and health behaviors were analyzed with single and multivariate binary logistic regression. The model was adjusted by the variables of all groups, adopting a 5% significance level. The values of odds ratio (OR) and respective confidence intervals were obtained.

**RESULTS:**

Women (OR = 0.74; 95%CI 0.66–0.82) had a lower chance of exposure to chemicals. The highest chances were observed in groups with no instruction or that attended up to middle-school (OR = 1.77; 95%CI 1.50–2.08), high school (OR = 1.62; 95%CI 1.37–1.91), age between 25 and 54 years (OR = 1.26; 95%CI 1.07–1.48), current smokers (OR = 1.21; 95%CI 1.07–1.37), who reported tiredness (OR = 1.35; 95%CI 1.21–1.50), hearing difficulties (OR = 1.24; 95%CI 1.04–1.48) and who reported having suffered an accident at work (OR = 2.00; 95%CI 1.57–2.54).

**CONCLUSIONS:**

The unprecedented results cover the entire workforce. Positive associations with hearing loss, smoking and history of work accidents are consistent, as well as the inverse association with education level and gender differences. The absence of association with asthma was surprising. To fill gaps in investigations on chronic non-communicable diseases, we suggested improving the PNS collection instrument in the occupational dimension.

## INTRODUCTION

In the ranking of risks related to the global burden of disease in Brazil, in 2015, occupational factors presented a prominent position when compared with other factors such as insufficient physical activity among men and alcohol use among women^1^. Exposure to chemical risks in the work environment has not been sufficiently characterized and dimensioned in Brazil. Technological innovations incorporated into the systems and processes increased the number of chemicals manufactured, in addition to the dissemination of by-products during their manufacture^2^. The world’s distribution of the production and consumption of chemicals is heterogeneous, generating inequalities in exposure to harmful agents. Lately, the participation of the BRICS countries, Brazil included, in the annual sales of chemicals increased from 13% to 28%, whereas the participation of European Union countries^3^decreased from 77% to 63%. In 2010, the Dominican Republic imported more than 6,000 tons of pesticides, of which 50% were banned in European Union countries^4^.

Exposure to particles disseminated in the workplace contributes to 15% of chronic obstructive pulmonary diseases^5^. It is estimated that 2% to 8% of all cancers are caused by agents present in the work environment, mostly chemicals. These chemicals include arsenic, asbestos, beryllium benzene, cadmium, chromium, nickel, diesel, ethylene oxide, ionizing radiation and silica, among others, widely used in various productive sectors^2^. The impact of occupational risk factors on cancer in Brazil was 2.3% among men and 0.3% among women. These numbers are lower than in other countries. The authors suggest an underestimated prevalence in Brazil, where estimates use official sources^6^. However, the informal labor market, which is hardly covered by information systems, absorbs more than 50% of the workforce and tends to concentrate more unhealthy positions and functions than the formal market^7,8^. Moreover, cases tend to focus on small groups of workers, which are largely not included in official statistics^9,10^.

Since the 1970s, several countries have started conducting periodic surveys on working conditions and health in national or transnational samples of workers to identify occupational exposure to chemicals, among other objectives^11^. Between 2007 and 2012, this initiative was incorporated by Latin American countries, which collected data in the workplace or at the worker’s household through self-reports^10^. Specific tests showed good reliability of the questionnaires^12^.

In Brazil, the results of the national survey of the Brazilian Institute of Geography and Statistics (IBGE) on employment are periodically published, in addition to those originated from national surveys on the health of the general population conducted by the Ministry of Health and partner institutions^13^. The first does not incorporate specific modules on health. The latter do not incorporate modules on employment and work. In 2013, the National Health Survey inaugurated the possibility of broadening the perspective, once the E module on the employment situation of the adult drawn for the interview at home and module M on handling chemicals by this adult in the workplace were incorporated, as well as specific modules for morbidities, risk behaviors and access to services^14^. This information is useful because it covers gaps in official sources. In addition to this advantage, the National Health Survey covers adults regardless of the type of insertion in the workforce. Surveys with instruments applied in households through interviews with workers have been proposed^12,15^.

Despite its limits, the National Health Survey already show encouraging results, such as monitoring risk behaviors^16^, access to services^17^ and risks of cardiovascular diseases^18^, to name a few.

We sought to describe the associated factors and the prevalence of self-reported exposure to chemical substances at work in the sample of Brazilian active adults, according to PNS data.

## METHODS

### Data Source and Target Population

The PNS is a population-based household survey that is part of the Integrated System of Household Surveys (SIPD) of IBGE, in which the entire national territory was included. The target population of the PNS consists of people living in permanent private households (PPH), and the questionnaire applied in the selected households consists of three parts: household information; general characteristics of all its residents; and information from an adult resident, 18 years of age or older, randomly selected. This last part provided most of the information of interest in our study.

### PNS Sample Planning

The primary sampling units (PSU) of the PNS are census tracts or sets of census tracts, when they have few households. PSU were stratified according to four different criteria: administrative, geographic, situation and statistical. The sampling plan used was cluster sampling in three stages of selection, and in the first stage the selection of the census tract, in the second stage the selection of the household and in the third stage the selection of a resident aged 18 years or more within each household of the sample. In Souza-Júnior’s work^19^, detailed information on the PNS sample design is available.

Our sample consisted of adults aged 18 years or older that answered question E1 of module E: “In the week of July 21–27, 2013 (reference week), did you work as regular employee or intern for at least an hour in any activity paid with cash?”. Our sample has 36,442 Brazilians that responded positively to E1 and, therefore, are considered active.

### Study variables

Module M of the PNS questionnaire is called “Other characteristics of work and social support.” Question M11 is divided into eight questions related to occupational exposure (chemical, physical and psychosocial risks), as follows: handling of chemical substances, handling of municipal waste (garbage); exposure to noise (loud noise), involvement in activities that lead to nervousness; long exposure to the sun; exposure to biological material; handling of radioactive material; exposure to industrial dust (marble dust). The individual that responded positively to the item “handling of chemical substances” was considered to be exposed to chemicals: “Thinking about your work, are you exposed to any of these factors that may affect your health?” Among the alternatives, two items refer to chemical agents: a) handling of chemical substances; and h) exposure to industrial dust (marble dust). The first was used because it coincides with the question tested in the instrument *Cuestionario Básico sobre Condiciones de Trabajo, Empleo y Salud en América Latina y el Caribe* (CTESLAC questionnaire). This questionnaire is a consensus among researchers from several countries. The test results indicated good reliability of the question^12^. The second item refers to marble powder. However, since it refers to a specific agent, it was not used, given the purpose of this research to know the exposure to chemical substances in general.

In the first group of explanatory variables, the following sociodemographic factors were analyzed: gender (male and female), age group (18 to 24 years, 25 to 54 years and 55 years or more), marital status (with partner and without partner), level of education (college, high and middle school or no instruction), race (white, black, brown and others). In the second, the factors related to health and life habits: health assessment (“very good,” “good” and “regular,” “bad” or “very bad”), sleep problems (“no” and “yes”), tiredness (“no” and “yes”), work accident in the prior 12 months (“no” and “yes”), diagnosis of hypertension (“no” and “yes”), diagnosis of depression (“no” and “yes”), diagnosis of asthma or asthmatic bronchitis (“no” and “yes”), heavy episodic drinking – considered as the intake of four doses or more on a single occasion (“no” and “yes”), and current smoking (“no” and “yes”). The question that gave rise to the variable hearing impairment [“Do you have hearing impairment?” (“no” and “yes”)] followed the introduction: “Now let’s address permanent hearing loss, that is, partial or total loss of the possibilities of hearing.”

### Statistical Analysis

First, a descriptive analysis of the outcome “exposure to chemicals” was performed by stratifying the sample per federation unit (FU), and this distribution was illustrated by constructing a thematic map using the Mapi-info program, version 10.0. Initially, a descriptive analysis of all studied variables was made by estimating relative frequencies and constructing a bar graph. The evaluation of the possible factors associated with exposure to chemical substances was performed using the single and multivariate binary logistic regression model. The significance level adopted for the selection of variables in the univariate analysis was 20%. The variables that reached this level were included in the multivariate analysis. Each block of variables (sociodemographic, morbidity-related and behavior-related characteristics) created a model to remove the variables using the backward method. The variables at 5% significance level continued in the final model of each group.

Then the model was adjusted by the variables of all groups, keeping the significant ones at the level of 5%. The odds ratio (OR) value were estimated, with 95% confidence interval (95%CI), both in the univariate and multivariate analyses. Hosmer-Lemeshow test and prediction calculation qualified the model adjustment. The probabilities of occurrence of the outcome were also estimated according to different profiles, considering the equation of the final logistic model.

All analyses were performed with attention to the characteristics of the complex sample of the PNS, considering the expansion factors or sample weights of the households and all their residents, as well as of the resident selected for the interview. The strata used in the sampling plan were also defined. The sample expansion was performed through the svy command, available in the Stata version 12.0 program, and it was considered in all analyses performed.

The National Research Ethics Commission (*Conep*) was responsible for the approval of the PNS project, through process no. 328,159, on June 26, 2013.

## RESULTS

**Figure 1:**
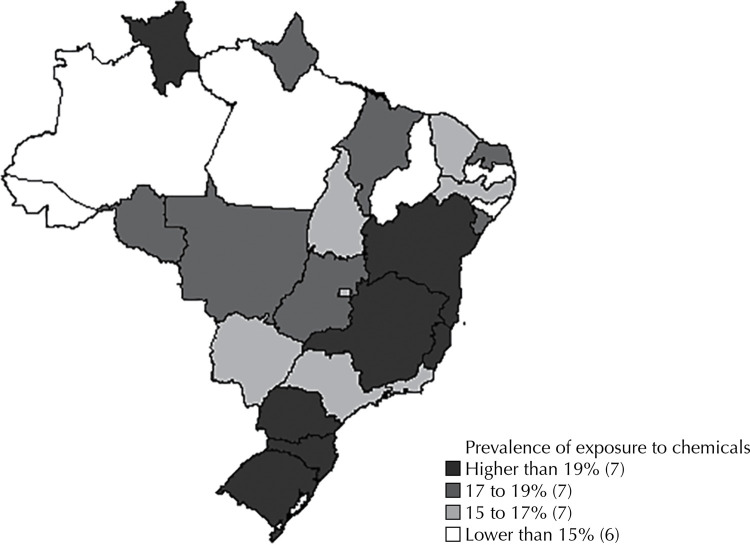
Prevalence of exposure to chemical substances in the Brazilian population in each of the 26 states and in the Federal District, according to data from the National Health Survey, Brazil, 2013.

The prevalence of exposure to chemical substances in the Brazilian population was 18.1%. Figure 1 shows the distribution of prevalence in Brazil, in which the South region stood out, with its three states showing the highest percentages.

The sample consisted of 53.4% of men. The prevalence of exposure to chemicals was 20.3% among men and 15.5% among women (data not shown in table). Regarding the characteristics of the sample, 74.5% reported age between 25 and 54 years, 60.2% had no partners, 41.5% reported having attended up to middle-school or having no instruction and 48.5% were mixed-race (Table 1).

**Table 1:** Univariate analysis evaluating the sociodemographic factors associated with exposure to chemical substances, according to data from the National Health Survey, Brazil, 2013.

	Percentage total sample (%)	Prevalence of exposure to chemicals (%)	p*	OR (95%CI)
Gender				
Male	53.4	20.3	< 0.001	1.00
Female	46.6	15.5		0.72 (0.65–0.80)
Age group (years)				
18–24	12.1	15.9	0.001	1.00
25–54	74.5	19.1		1.25 (1.07–1.47)
≥ 55	13.4	15.6		0.98 (0.80–1.19)
Marital status				
With a partner	39.8	18.9	0.070	1.00
No partner	60.2	17.5		0.94 (0.83–1.01)
Education level				
College	22.5	12.2	< 0.001	1.00
High school	35.9	18.7		1.64 (1.39–1.94)
Middle-school or @no instruction	41.5	20.9		1.90 (1.62–2.23)
Race				
White	40.5	17.8	0.178	1.00
Black	9.5	20.6		1.20 (1.00–1.44)
Mixed race	48.5	18.1		1.02 (0.92–1.14)
Others	1.5	15.9		0.88 (0.56–1.37)

OR: odds ratio; 95%CI: 95% confidence interval. Values with statistical significance are presented in bold.

* Pearson’s chi-square test.

Regarding health-related factors, 27.8% rated their health as regular, bad or very bad, 27.2% reported having sleep problems and 31.0% reported tiredness. The prevalence of reporting hearing difficulties was 6.8%. Regarding work accidents, 2.9% said they had suffered some. Out of the interviewees, 16.6% reported having been diagnosed with hypertension, 5.9% with depression and 4.3% with asthma or asthmatic bronchitis. Regarding life habits, 57.0% of the interviewees have alcohol consumption considered at risk and 15.4% currently smoke (Table 2).

**Table 2:** Univariate analysis evaluating the characteristics related to the health situation and life habits associated with exposure to chemical substances, according to data from the National Health Survey, Brazil, 2013.

	Percentage total sample (%)	Prevalence of exposure to chemicals (%)	p*	OR (95%CI)
Self-assessment of health				
Very good	15.0	16.7	**0.015**	1.00
Good	57.3	17.8		1.08 (0.94–1.23)
Regular, poor and very poor	27.8	19.7		1.22 (1.05–1.43)
Sleep problems				
No	72.8	17.2	**< 0.001**	1.00
Yes	27.2	20.8		1.26 (1.14–1.40)
Tiredness				
No	69.0	16.8	**< 0.001**	1.00
Yes	31.0	21.2		1.33 (1.20–1.47)
Hearing difficulty				
No	93.2	17.8	**< 0.001**	1.00
Yes	6.8	22.9		1.38 (1.16–1.63)
Suffered an accident at work in the prior 12 months				
No	97.1	17.7	**< 0.001**	1.00
Yes	2.9	33.2		2.32 (1.83–2.93)
Arterial hypertension				
No	83.4	18.1	0.422	1.00
Yes	16.6	18.8		1.05 (0.93–1.19)
Diagnosed with depression				
No	94.1	17.9	**0.013**	1.00
Yes	5.9	21.6		1.26 (1.05–1.52)
Diagnosed with asthma or asthmatic bronchitis				
No	95.7	18.1	0.252	1.00
Yes	4.3	20.1		1.15 (0.91–1.44)
Heavy episodic drinking				
No	43.0	20.5	0.385	1.00
Yes	57.0	21.6		1.07 (0.92–1.25)
Current smoker				
No	84.6	17.4	**< 0.001**	1.00
Yes	15.4	22.4		1.37 (1.22–1.55)

OR: odds ratio; 95%CI: 95% confidence interval. Values with statistical significance are presented in bold.

* Pearson’s chi-square test.

According to the univariate analysis, sociodemographic factors (Table 1) associated with higher prevalence of chemical exposure were: female gender, age ranging between 25–54 years, living without a partner, and higher education level (p < 0.05). Regarding the factors related to health (Table 2), those that were significantly associated with the higher prevalence of exposure to chemical substances were: sleep problems, tiredness and hearing difficulties. They were also significantly associated with a higher prevalence of exposure to chemicals, having assessed health as regular, bad or very bad; having suffered an accident at work and reporting a diagnosis of depression. Considering life habits shown in Table 2, being a current smoker was associated with a higher prevalence of exposure to chemical substances.

In the final model, the variables gender, age group, level of education, current smoking, tiredness, hearing difficulties and work accidents remained associated with exposure to chemical substances (Table 3). The model presented good adjustment according to Hosmer-Lemeshow statistics at a 5% significance level.

**Table 3:** Multivariate analysis evaluating sociodemographic factors, life habits and health situation associated with exposure to chemical substances, according to data from the National Health Survey, Brazil, 2013.

	OR (95%CI)			
	Model group 1 – Sociodemographic characteristics	Model group 2 – Lifestyles	Model group 3 – health situation	Final model
Gender				
Male	1.00			1.00
Female	0.75 (0.68–0.84)			0.74 (0.66–0.82)
Age group (years)				
18–24	1.00			1.00
25–54	1.28 (1.09–1.50)			1.26 (1.07–1.48)
≥ 55	0.92 (0.75–1.13)			0.92 (0.75–1.13)
Education level				
Higher education	1.00			1.00
High school	1.63 (1.38–1.92)			1.62 (1.37–1.91)
Middle-school or no instruction	1.86 (1.58–2.18)			1.77 (1.50–2.08)
Current smoker				
No		1.00		1.00
Yes		1.37 (1.22–1.55)		1.21 (1.07–1.37)
Tiredness				
No			1.00	1.00
Yes			1.28 (1.16–1.43)	1.35 (1.21–1.50)
Hearing difficulty				
No			1.00	1.00
Yes			1.29 (1.08–1.53)	1.24 (1.04–1.48)
Work accident				
No			1.00	1.00
Yes			2.22 (1.75–2.82)	2.00 (1.57–2.54)

OR: odds ratio; 95%CI: 95% confidence interval

Note: Final model adjustment p-value = 0.336

According to multivariate analysis, women (OR = 0.74; 95%CI 0.66–0.82) have a lower chance of exposure to chemicals than men. A higher chance of this exposure was observed in the group aged between 25 and 54 years (OR = 1.26; 95%CI 1.07–1.48) when compared with those aged up to 25 years, and in those that reported having studied up to the middle-school or no instruction (OR = 1.77; 95%CI 1.50–2.08) when compared with those with higher education. Individuals with high school (OR = 1.62; 95%CI 1.37–1.91) also had a higher chance of exposure to chemicals when compared with those with higher education. Smokers (OR = 1.21; 95%CI 1.07–1.37), with tiredness (OR = 1.35; 95%CI 1.21–1.50), hearing difficulties (OR = 1.24; 95%CI 1.04–1.48) and that reported having suffered an accident at work (OR = 2.00; 95%CI 1.57–2.54) had a higher chance of exposure to chemical substances when compared with those with an antagonistic condition (Table 3).

**Figure 2:**
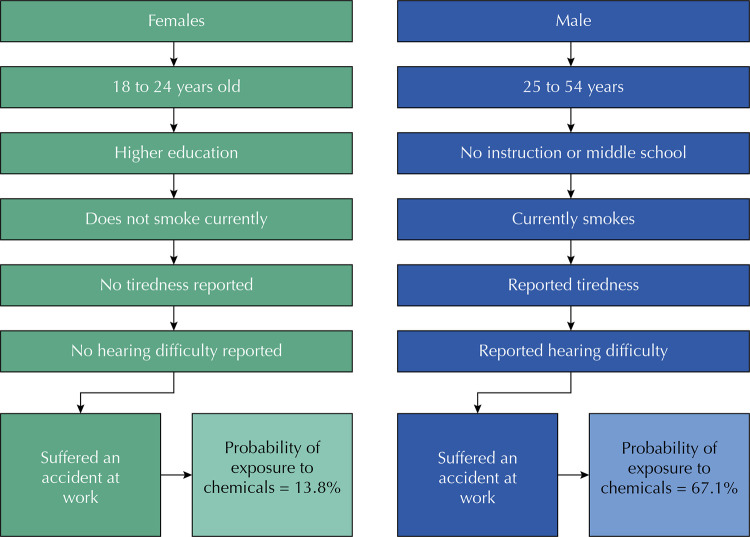
Probability diagram of exposure to chemical substances, according to the final logistic regression model, with data from the National Health Survey (PNS), Brazil, 2013.

The prevalence of exposure to chemical substances was estimated considering the final model of binary logistic regression regarding the profiles shown in Figure 2. The probability of exposure to chemical substances was 13.8% among women aged between 18 and 24 years, with higher education, who do not currently smoke, who did not report tiredness or hearing difficulties and did not suffer an accident at work in the prior 12 months. On the other hand, we observed an increase of 67.1% in the probability of exposure in the group of men aged between 25 and 54 years, with no instruction or who attended up to middle-school, who currently smoke and reported tiredness or hearing difficulties and who suffered work accidents in the prior year.

## DISCUSSION

According to the data collected at the household, almost one-fifth of Brazilian workers, regardless of employment or type of remuneration, reported handling of chemical substances at work. Although high, the occurrence is below the statistics recorded in the United States and France: 22.3%^20^ and 30%^21^, respectively. When men and women are observed separately, the results are above the recorded in Honduras and Argentina, but below what was found in Ecuador^22^. Positive associations with hearing loss, smoking and history of work accidents, as well as the inverse association with education level and gender differences, are consistent. The absence of association with diagnosis of asthma or asthmatic bronchitis was, to some extent, unexpected.

The higher occurrence of occupational exposure among men was predictable, since there are more men absorbed in sectors in which handling or contact with chemical substances predominate. The configurations of male and female identities are reproduced in the distribution of men and women in the labor market.

Regional differences were observed in the exposure to chemical substances, indicating an association with the geoeconomics in the country. The self-report of exposure was almost threefold in states in the South region when compared with those in the Northeast region. Clarifications about the regional heterogeneity of Brazilian industry are useful to interpret this result. In the 1990s, numerous industrial companies moved or settled in the Southern region, which is close to Mercosur countries, probably attracted by good infrastructure, wages lower than those in the Southeast region and recognized performance in the fiscal war^24^. When analyzing the most important federation units for each economic activity, the authors found the strength of Minas Gerais and São Paulo for industry, followed, without distinction between them, by the states of Ceará and Rio de Janeiro, in addition to the three southern states^25^. This approximates with the map of exposure to chemicals. Production sectors should be further studied with finer levels of disaggregation^26^. The new scenario of production, consumption and configuration of the labor market probably brings elements to interpret the higher occurrence of exposure in regions considered less industrialized, because conservation and cleaning sectors and activities in agro-industry abundantly incorporate chemical substances and are present in all regions.

The association with the report of hearing difficulty show the necessity to understand the ototoxic effect of various chemical substances potentially capable of causing sensorineural alterations in the ear. Metal solvents and organophosphate pesticides are included among the main chemical agents that can lead to hearing alterations^26^.

Regarding the association with occupational accidents, combined exposure to various risks is recognized, especially in the industry^27^. Most chemicals handled in work processes cause neurological effects, which decrease concentration and wakefulness^28^. It is plausible that there is a relationship between this effect and the occurrence of accidents. Moreover, more polluted environments coincide with those at greater risk for accidents. Such findings justify the interest in addressing a combined contribution from occupational risks.

The incidence of asthma in adults is often related to occupational exposure, being, for example, the most common health problem among workers in the furniture industry in Turkey^29^. Asthma is considered an occupational disease when a relationship between its symptoms and substances present in the work environment is identified. Episodes are induced by sensitivity to a specific substance inhaled by the worker. Another frequent picture concerns the worsening of pre-existing asthma by a stimulus present in the workplace^30^. The absence of association observed in the PNS sample is probably an effect of the selection bias^31^, since people intervene in their own situations, for example, changing jobs or avoiding the handling of chemicals to prevent respiratory symptoms. Limits related to the coverage of health systems are possible, since the questionnaire mentioned medical diagnosis.

Positive association with tiredness was expected. Painters, varnishers, attendants and farmers are often exposed to the organic solvents that compose the products used in the development of their tasks. Many symptoms related to these substances are nonspecific, including nausea, difficulty concentrating and attention, sleep disorders, fatigue and abnormal tiredness^28^. In Turkey, 39.8% of workers in the furniture industry, whose environments are generally contaminated by the by-products of paints and varnishes, complained of abnormal fatigue^29^. In coffee plantations in the Dominican Republic, 76% of workers exposed to various classes of pesticides reported abnormal tiredness, as opposed to 39% of those not exposed^4^.

Generally, less educated workers occupy more dangerous jobs. Thus, this is the reverse law of risk: the overlap of harmful factors tends to be inversely proportional to the stock of resources and the degree of power of the individuals and groups affected to modify their situation^20^. The lack of safety information, mistakes in spray operations and weak personal protection devices, which are related to the low education of workers, are among the aggravating factors of the effects of exposure to chemicals^6^. Moreover, low schooling also has to do with fewer resources for the elaboration of positive confrontations, from which elements are obtained to interpret the adoption of less healthy habits – in this case, smoking.

Significant associations between smoking habits and occupational exposure to chemicals are consistent. Blood levels of substances generated in the work environment are higher in smokers than in their non-smoking colleagues^29^. Previous studies have evidenced the greater chance of individuals exposed to unhealthy work environments to report cigarette consumption as a negative way to deal with stress originated in these situations^32^. PNS 2013 data allowed the authors to observe the reduction in the prevalence of tobacco smokers in the analyzed period (2008 to 2013)^33^. However, gender disparities persist, with less reduction among men. We can assume the influence of working conditions in this area, since more polluted posts allocate more men than women^23^.

Regarding occupational hygiene, the decrease in the magnitude of exposure to chemical substances in the workplace has been reported. Probably, the advance of worker health programs in the control of exposure contributes to this reality, in addition to the reduction of the effective in traditional and dangerous industries, such as coal mining, iron and steel casting, mineral extraction, etc. Overall, they would be a reflection of the change in the occupational structure that is expressed on three fronts: a decrease in the agricultural workforce; decreasing trend at the occupational level in industry, construction and mining; and expansion of labor absorption in the service sector^2^. This reality led to the favor of other occupational health priorities, such as stress at work, accidents due to falls and musculoskeletal problems. On the other hand, in sectors whose greatest expression in the absorption of the workforce has occurred recently, workers are routinely exposed to chemicals (e.g., leisure and entertainment industry and conservation and cleaning companies). Admittedly relevant, either due to the magnitude or due to the aggressiveness of the effects of these substances on work capacity, recent data on disease load and fraction attributable to occupational risks for cancer^6^ in the Brazilian population reinforce the importance of continuing to try to identify occupational exposure.

In the PNS questionnaire, only one question asked about exposure to chemicals.

Although the content of the question coincides with the filter question of most instruments tested and used in recent national and transnational surveys focused on samples of workers^15^, information biases are expected from self-reports obtained from face-to-face interviews^10^.

The researchers’ recent effort to standardize questions about working conditions to encourage periodic surveys in Latin American countries and increase the possibility of comparing was successful^34^. The CTESLAC questionnaire indicaste tio questiones for the topic “chemicals”: Q27 *Handles, applies or is in contact with chemical/harmful/toxic substances*? and Q28. *Breathes chemical substances in the form of dust, fumes, aerosols, vapors, gases and/or fog* (*excluding tobacco smoke*)?^15^. The impasses to create consensus in the research instruments structure international networks of researchers. The question of the PNS questionnaire coincides with one of the questions of the questionnaire already applied in Latin American countries and the European Union. However, an effort is expected to improve the questionnaire to complete the questions and favor the desired consensus.

The lack of reliable and quality information encourages researchers to seek self-reported information on a wide variety of exposures, such as medication use, diet, smoking and occupational history. Using self-reports to define exposed and unexposed and to estimate the duration of exposure is one of the critical components of most epidemiological studies. However, results of specific studies, such as the verification of the quality of self-reported information on exposure to pesticides, encouraged researchers to maintain and apply questionnaires through interviews^35^.

Unlike information on diseases and accidents, which are also essential, studies on exposure have the advantage of facilitating timely and effective intervention, since they precede the effects and damage. We expect our results to instigate the (re)formulation of questions about occupational exposure in future national health surveys, because the various existing information systems are fragmented, usually incomplete, either because they do not cover the population informally inserted in the labor market, or because they depend on systems not yet consolidated^6^.

Possibly, the results of the PNS on the self-report of exposure to chemicals will be valuable information for the health surveillance systems of workers, besides providing clues to the investigations on risk factors for the burden of chronic-degenerative diseases. Finally, our results reinforce previous indications regarding the relevance of considering the characteristics of employment and work in the programs aimed at smoking cessation in the population^32^.

## CONCLUSIONS

Although possibly underestimated, the occurrence of self-reported exposure to chemical substances in the study was high. Except for the absence of association with asthma or asthmatic bronchitis, in which coverage problems should be considered, the positive associations with tiredness, hearing difficulties, work accidents and smoking are consistent, as well as the inverse association with the level of education and gender differences. Despite being in convergence with a panorama recently described in the countries of Latin America and the Caribbean, the information bias according to the content and format of the issue that guided the construction of the main variable limits the interpretation of the results. We suggested the improvement of the PNS collection instrument in the occupational dimension to fill gaps in investigations on chronic non-communicable diseases.
