# Supplementary material for: Factors associated with self-reported exposure to chemical substances at work in Brazil: results from the National Health Survey, 2013
Source: Rev Saude Publica. 2020 Aug 28;54:92. doi: 10.11606/s1518-8787.2020054001461 (PMC7454166; doi:10.11606/s1518-8787.2020054001461)
Supplement: Translation

# Translation: Exposição a agentes químicos no trabalho no Brasil: resultados da Pesquisa Nacional de Saúde, 2013

**Authors:** Ada Ávila Assunção, Mery Natali Silva Abreu, Priscila Sílvia Nunes Souza

**Affiliations:** I Brasil Universidade Federal de Minas Gerais. Faculdade de Medicina. Departamento de Medicina Preventiva e Social. Belo Horizonte, MG, Brasil; II Brasil Universidade Federal de Minas Gerais. Escola de Enfermagem. Departamento de Gestão em Saúde. Belo Horizonte, MG, Brasil; III Brasil Universidade Federal de Minas Gerais. Escola de Enfermagem. Belo Horizonte, MG, Brasil

**Keywords:** Exposição Ocupacional, Compostos Químicos, Condições de Trabalho, Saúde do Trabalhador, Inquéritos Epidemiológicos, Pesquisa Nacional de Saúde

## Abstract

**OBJETIVO:**

Descrever os fatores associados e a prevalência de exposição autorrelatada a substâncias químicas no trabalho em uma amostra de adultos brasileiros que participaram da Pesquisa Nacional de Saúde, realizada entre 2013 e 2014.

**MÉTODOS:**

A amostra para análise da exposição foi constituída por trabalhadores com 18 anos ou mais de idade que responderam à questão E1 do módulo E: “Na semana de 21 a 27 de julho de 2013 (semana de referência), você trabalhou ou estagiou, durante pelo menos uma hora, em alguma atividade remunerada em dinheiro?” Os dados sociodemográficos, situação e comportamentos de saúde foram analisados com regressão logística binária uni e multivariada. O modelo foi ajustado pelas variáveis de todos os blocos, adotando-se o nível de significância de 5%. Obtiveram-se os valores de *odds ratio* (OR) e respectivos intervalos de confiança.

**RESULTADOS:**

Mulheres (OR = 0,74; IC95% 0,66–0,82) tiveram menor chance de exposição a substâncias químicas. As maiores chances foram observadas nos grupos com nível fundamental de instrução ou sem instrução (OR = 1,77; IC95% 1,50–2,08), nível médio de instrução (OR = 1,62; IC95% 1,37–1,91), idade entre 25 e 54 anos (OR = 1,26; IC95% 1,07–1,48), fumantes atuais (OR = 1,21; IC95% 1,07–1,37), com problemas de cansaço (OR = 1,35; IC95% 1,21–1,50), com dificuldade auditiva (OR = 1,24; IC95% 1,04–1,48) e que relataram ter sofrido acidente de trabalho (OR = 2,00; IC95% 1,57–2,54).

**CONCLUSÕES:**

Os resultados inéditos abrangem o conjunto da força de trabalho. Associações positivas com cansaço, dificuldade auditiva, acidentes de trabalho e tabagismo, assim como a associação inversa com o nível de escolaridade, além das diferenças de gênero, são consistentes. A ausência de associação com asma foi surpreendente. A fim de preencher lacunas nas investigações sobre doenças crônicas não transmissíveis em adultos, sugere-se aperfeiçoar o instrumento da Pesquisa Nacional de Saúde na dimensão ocupacional.

## INTRODUÇÃO

No ranking dos riscos relacionados à carga global de doença no Brasil, em 2015, os fatores ocupacionais apresentaram posição de destaque em relação a outros fatores como atividade física insuficiente no grupo dos homens e uso de álcool no grupo das mulheres^1^. A exposição aos riscos químicos no ambiente de trabalho não foi suficientemente caracterizada e dimensionada no Brasil. Inovações tecnológicas incorporadas aos sistemas e processos aumentaram o número de produtos químicos fabricados, além da disseminação de subprodutos durante a fabricação dos principais^2^. A distribuição mundial da produção e consumo das substâncias químicas é heterogênea, gerando desigualdades na exposição aos agentes nocivos. Nas últimas décadas, a participação dos países do bloco Brics, em que consta o Brasil, nas vendas anuais de químicos aumentou de 13% a 28%, enquanto diminuiu de 77% a 63% a venda realizada por países da União Europeia^3^. Em 2010, a República Dominicana importou mais de 6.000 toneladas de pesticidas, dos quais 50% estavam proibidos nos países da União Europeia^4^

A exposição às partículas disseminadas no ambiente laboral contribui para 15% das doenças pulmonares obstrutivas crônicas^5^. Estima-se que 2% a 8% de todos os cânceres sejam causados por agentes presentes no ambiente laboral, produtos químicos em sua maioria. Entre estes, estão o arsênico, amianto, benzeno berílio, cádmio, cromo, níquel, diesel, óxido de etileno, radiação ionizante e sílica, entre outros, utilizados largamente em vários setores produtivos^2^. O impacto dos fatores de risco ocupacionais sobre o câncer no Brasil foi de 2,3% em homens e 0,3% em mulheres. Esses números são inferiores àqueles identificados em outros países. Os autores sugerem ter havido uma subestimação da prevalência no Brasil, onde o cálculo das estimativas utiliza fontes oficiais^6^. Ora, o mercado informal de trabalho, dificilmente coberto pelos sistemas de informação, além de absorver mais de 50% da mão de obra, tende a concentrar cargos e funções mais insalubres do que se observa no mercado formal^7,8^. Além disso, os casos tendem a se concentrar em pequenos grupos de trabalhadores, que em grande parte não são incluídos nas estatísticas oficiais^9,10^.

Desde os anos 1970, vários países iniciaram a realização periódica de inquéritos sobre condições laborais e saúde em amostras nacionais ou transnacionais de trabalhadores a fim de, entre outros objetivos, identificar a exposição ocupacional a substâncias químicas^11^. Entre 2007 e 2012, essa iniciativa foi incorporada por países latino-americanos, que colheram dados nos locais de trabalho ou no domicílio do trabalhador por meio de autorrelatos^10^. Testes específicos evidenciaram boa confiabilidade dos questionários^12^.

No Brasil, periodicamente são divulgados os resultados da pesquisa nacional do Instituto Brasileiro de Geografia e Estatística (IBGE) sobre emprego, além daqueles originados dos inquéritos nacionais sobre a saúde da população geral realizados pelo Ministério da Saúde e instituições parceiras^13^. A primeira não incorpora módulos específicos sobre saúde. As últimas não incorporam módulos sobre emprego e trabalho. Em 2013, a Pesquisa Nacional de Saúde inaugurou a possibilidade de ampliar a perspectiva, uma vez incorporados o módulo E sobre a situação no emprego do adulto sorteado para a entrevista no domicílio e o módulo M sobre manuseio de substâncias químicas por esse adulto no local de trabalho, além de módulos específicos para morbidades, comportamentos de risco e acesso a serviços^14^. Essas informações são úteis, pois cobrem lacunas das fontes oficiais. Além dessa vantagem, a Pesquisa Nacional de Saúde abrange adultos independentemente do tipo de inserção na força de trabalho. Vale destacar ainda que têm sido indicadas as propostas de inquéritos com instrumentos aplicados nos domicílios por meio de entrevistas aos trabalhadores^12,15^.

Sobre a Pesquisa Nacional de Saúde, ainda que se reconheçam os seus limites, são animadores os resultados, que já produzem os seus frutos, como monitoramento de comportamentos de risco^16^, acesso a serviços^17^ e riscos de doenças cardiovasculares^18^, para citar alguns.

O objetivo foi descrever os fatores associados e a prevalência de exposição autorrelatada a substâncias químicas no trabalho na amostra de adultos brasileiros ocupados, de acordo com os dados da PNS.

## MÉTODOS

### Fonte dos Dados e População-Alvo

A PNS é um inquérito domiciliar de base populacional que faz parte do Sistema Integrado de Pesquisas Domiciliares (SIPD) do IBGE, no qual todo o território nacional foi incluído. A população-alvo da PNS é constituída pelas pessoas residentes em domicílios particulares permanentes (DPP), e o questionário aplicado nos domicílios selecionados é composto por três partes: informações do domicílio; características gerais de todos os seus moradores; e informações de um morador adulto, com 18 anos ou mais de idade, selecionado aleatoriamente. Essa última parte forneceu a maioria das informações de interesse no presente estudo.

### Planejamento Amostral da PNS

As unidades primárias de amostragem (UPA) da PNS são setores censitários ou conjuntos de setores censitários, quando eles contam com poucos domicílios. As UPA foram estratificadas segundo quatro diferentes critérios: administrativo, geográfico, de situação e estatístico. O plano amostral empregado foi o de amostragem por conglomerado em três estágios de seleção, sendo no primeiro estágio a seleção do setor censitário, no segundo estágio a seleção do domicílio e no terceiro estágio a seleção de um morador com 18 anos ou mais dentro de cada domicílio da amostra. No trabalho de Souza-Júnior^19^estão disponíveis informações detalhadas sobre o desenho amostral da PNS.

A amostra do presente estudo foi constituída por adultos com 18 anos ou mais de idade que responderam à questão E1 do módulo E: “Na semana de 21 a 27 de julho de 2013 (semana de referência), você trabalhou ou estagiou, durante pelo menos uma hora, em alguma atividade remunerada em dinheiro?” Responderam positivamente à E1 36.442 brasileiros que, portanto, são considerados ocupados e constituem a amostra analisada no presente estudo.

### Variáveis do Estudo

O módulo M do questionário da PNS é denominado “Outras características do trabalho e apoio social”. A questão M11 é desdobrada em oito perguntas atinentes à exposição ocupacional (riscos químicos, físicos e psicossociais), assim definidas: manuseio de substâncias químicas, manuseio de resíduos urbanos (lixo); exposição a ruído (barulho intenso), envolvimento em atividades que levam ao nervosismo; exposição longa ao sol; exposição a material biológico; manuseio de material radioativo; exposição a poeira industrial (pó de mármore). Foi considerado como exposto a substâncias químicas o indivíduo que respondeu positivamente ao item “manuseio de substâncias químicas” da pergunta: “Pensando em todos os seus trabalhos, o(a) sr(a) está exposto(a) a algum destes fatores que podem afetar a sua saúde?” Entre as alternativas, dois itens se referem a agentes químicos: a) manuseio de substâncias químicas; e h) exposição a poeira industrial (pó de mármore). O primeiro foi utilizado porque coincide com a pergunta testada no instrumento *Cuestionario Básico sobre Condiciones de Trabajo, Empleo y Salud en América Latina y el Caribe* (questionário CTESLAC). O referido questionário é um consenso de pesquisadores de vários países. Os resultados do teste indicaram boa confiabilidade da referida pergunta^12^. O segundo item se refere ao pó de mármore. Mas, por se referir a um agente específico, não foi utilizado, haja vista o propósito da presente pesquisa de conhecer a exposição às substâncias químicas em geral.

No primeiro bloco de variáveis explicativas, foram analisados os seguintes fatores sociodemográficos: sexo (masculino e feminino), faixa etária (18 a 24 anos, 25 a 54 anos e 55 anos ou mais), estado civil (com parceiro e sem parceiro), nível de instrução (superior, médio e fundamental ou sem instrução), raça (branca, preta, parda e outros). Já no segundo bloco foram estudados os fatores relacionados à saúde e hábitos de vida: avaliação de saúde (muito boa, boa e regular, ruim ou muito ruim), problemas de sono (não e sim), problema de cansaço (não e sim), acidente de trabalho nos últimos 12 meses (não e sim), diagnóstico de hipertensão (não e sim), diagnóstico de depressão (não e sim), diagnóstico de asma ou bronquite asmática (não e sim ), consumo de álcool de risco – *heavy episodic drinking* –, considerado como a ingestão de quatro doses ou mais em uma única ocasião (não e sim), e fumar atualmente (não e sim). A pergunta que deu origem à variável deficiência auditiva [“Tem deficiência auditiva?” (não e sim)] seguiu a chave: “Agora vamos abordar a deficiência auditiva permanente, isto é, perda parcial ou total das possibilidades de ouvir”.

### Análise Estatística

Primeiramente, foi realizada uma análise descritiva do desfecho exposição a produtos químicos estratificando a amostra por unidade da federação (UF), e essa distribuição foi ilustrada por meio da construção de um mapa temático utilizando o programa Mapi-info versão 10.0. Subsequentemente, foi realizada análise descritiva de todas as variáveis de interesse por meio do cálculo das frequências relativas. A avaliação dos possíveis fatores associados à exposição a substâncias químicas foi realizada utilizando o modelo de regressão logística binária uni e multivariado. O nível de significância adotado para seleção das variáveis na análise univariada foi de 20%. As variáveis que alcançaram esse nível foram incluídas na análise multivariada. A cada bloco de variáveis (características sociodemográficas, características relacionadas à morbidade e características relacionadas a comportamentos), foi criado um modelo, de maneira a retirar as variáveis por meio do método *backward*. Continuaram no modelo final de cada bloco as variáveis significativas ao nível de 5%.

Em seguida, o modelo foi ajustado pelas variáveis de todos os blocos, mantendo mais uma vez as significativas ao nível de 5%. Foram estimados os valores de *odds ratio* (OR) com intervalo de confiança de 95% (IC95%) tanto na análise univariada quanto na multivariada. A estatística de Hosmer e Lemeshow e o cálculo de predição qualificaram o ajuste do modelo. Foram calculadas ainda as probabilidades de ocorrência do desfecho segundo diferentes perfis, sendo considerada a equação do modelo logístico final.

Todas as análises foram realizadas com atenção às características da amostra complexa da PNS, considerando os fatores de expansão ou pesos amostrais dos domicílios e todos seus moradores, assim como do morador selecionado para a entrevista. Também foram definidos os estratos utilizados no plano amostral. A expansão amostral foi realizada por meio da utilização do comando svy, disponível no programa Stata versão 12.0, e foi considerada em todas as análises realizadas.

A Comissão Nacional de Ética em Pesquisa (Conep) foi responsável pela aprovação do projeto PNS, pelo processo nº 328.159, em 26 junho de 2013.

## RESULTADOS

A prevalência de exposição a substâncias químicas na população brasileira foi de 18,1%. A Figura 1 ilustra a distribuição das prevalências no território brasileiro. A região Sul se destacou, com os seus três estados apresentando os maiores percentuais.

**Figura 1:** Prevalência de exposição a substâncias químicas na população brasileira em cada um dos 26 estados e no Distrito Federal, segundo dados da Pesquisa Nacional de Saúde, Brasil, 2013.

A amostra foi composta por 53,4% de homens, e a prevalência de exposição a substâncias químicas foi de 20,3% entre eles e de 15,5% entre as mulheres (dados não apresentados em tabela). Sobre as características da amostra, 74,5% informaram idade entre 25 e 54 anos, 60,2% não tinham parceiros, 41,5% informaram grau de instrução fundamental ou sem instrução e 48,5% eram pardos (Tabela 1).

**Tabela 1:** Análise univariada avaliando os fatores sociodemográficos associados à exposição a substâncias químicas, segundo dados da Pesquisa Nacional de Saúde, Brasil, 2013.

	Percentual da amostra total (%)	Prevalência de exposição a substâncias químicas (%)	p*	OR (IC95%)
Sexo				
Masculino	53,4	20,3	**< 0,001**	1,00
Feminino	46,6	15,5		0,72 (0,65–0,80)
Faixa etária (anos)				
18–24	12,1	15,9	**0,001**	1,00
25–54	74,5	19,1		1,25 (1,07–1,47)
≥ 55	13,4	15,6		0,98 (0,80–1,19)
Estado civil				
Com parceiro	39,8	18,9	0,070	1,00
Sem parceiro	60,2	17,5		0,94 (0,83–1,01)
Nível de instrução				
Superior	22,5	12,2	**< 0,001**	1,00
Médio	35,9	18,7		1,64 (1,39–1,94)
Fundamental ou sem instrução	41,5	20,9		1,90 (1,62–2,23)
Raça				
Branca	40,5	17,8	0,178	1,00
Preta	9,5	20,6		1,20 (1,00–1,44)
Parda	48,5	18,1		1,02 (0,92–1,14)
Outros	1,5	15,9		0,88 (0,56–1,37)

OR: *odds ratio*; IC95%: intervalo de confiança de 95%. Valores com significância estatística mostrados em negrito.

* Teste qui-quadrado de Pearson

Quanto aos fatores relacionados à saúde, 27,8% avaliaram sua saúde como regular, ruim ou muito ruim, 27,2% afirmaram ter problemas de sono e 31,0% afirmaram ter problemas de cansaço. A prevalência de relato de dificuldade auditiva foi de 6,8%. Sobre acidentes de trabalho, 2,9% afirmaram ter sofrido algum. Dos entrevistados, 16,6% afirmaram ter sido diagnosticados com hipertensão arterial, 5,9% com depressão e 4,3% com asma ou bronquite asmática. Quanto aos hábitos de vida, 57,0% dos entrevistados informaram consumo de álcool considerado de risco e 15,4% fumam atualmente (Tabela 2).

**Tabela 2:** Análise univariada avaliando as características relacionadas à situação de saúde e hábitos de vida associados à exposição a substâncias químicas, segundo dados da Pesquisa Nacional de Saúde, Brasil, 2013.

	Percentual da amostra total (%)	Prevalência de exposição a substâncias químicas (%)	p*	OR (IC95%)
Autoavaliação de saúde				
Muito boa	15,0	16,7	**0,015**	1,00
Boa	57,3	17,8		1,08 (0,94–1,23)
Regular, ruim e muito ruim	27,8	19,7		1,22 (1,05–1,43)
Problemas de sono				
Não	72,8	17,2	**< 0,001**	1,00
Sim	27,2	20,8		1,26 (1,14–1,40)
Problema de cansaço				
Não	69,0	16,8	**< 0,001**	1,00
Sim	31,0	21,2		1,33 (1,20–1,47)
Dificuldade auditiva				
Não	93,2	17,8	**< 0,001**	1,00
Sim	6,8	22,9		1,38 (1,16–1,63)
Sofreu acidente de trabalho nos últimos 12 meses				
Não	97,1	17,7	**< 0,001**	1,00
Sim	2,9	33,2		2,32 (1,83–2,93)
Hipertensão arterial				
Não	83,4	18,1	0,422	1,00
Sim	16,6	18,8		1,05 (0,93–1,19)
Diagnosticado com depressão				
Não	94,1	17,9	**0,013**	1,00
Sim	5,9	21,6		1,26 (1,05–1,52)
Diagnosticado com asma ou bronquite asmática				
Não	95,7	18,1	0,252	1,00
Sim	4,3	20,1		1,15 (0,91–1,44)
Consumo de álcool de risco (*heavy episodic drinking*)				
Não	43,0	20,5	0,385	1,00
Sim	57,0	21,6		1,07 (0,92–1,25)
Fumante atual				
Não	84,6	17,4	**< 0,001**	1,00
Sim	15,4	22,4		1,37 (1,22–1,55)

OR: *odds ratio*; IC95%: intervalo de confiança de 95%. Valores com significância estatística mostrados em negrito.

* Teste qui-quadrado de Pearson

À análise univariada, os fatores sociodemográficos (Tabela 1) associados à maior prevalência de exposição a substâncias químicas foram: ser do sexo masculino, ter idade na faixa de 25 a 54 anos e menor nível de instrução (p < 0,05). Com relação aos fatores relacionados à saúde (Tabela 2), aqueles que foram significativamente associados à maior prevalência de exposição a substâncias químicas foram: problemas de sono, problema de cansaço e dificuldade auditiva. Também foram significativamente associados à maior prevalência de exposição a substâncias químicas ter avaliado a saúde como regular, ruim ou muito ruim, ter sofrido acidente de trabalho e relatar diagnóstico de depressão. Ainda considerando a Tabela 2, dentre os hábitos de vida, ser fumante atual se mostrou associado à maior prevalência de exposição a substâncias químicas.

No modelo final, permaneceram associadas à exposição a substâncias químicas as variáveis sexo, faixa etária, nível de instrução, fumo atual, problemas de cansaço, dificuldade auditiva e acidente de trabalho (Tabela 3). O modelo apresentou bom ajuste, segundo a estatística de Hosmer e Lemeshow, ao nível de significância de 5%.

**Tabela 3:** Análise multivariada avaliando os fatores sociodemográficos, hábitos de vida e situação de saúde associados à exposição a substâncias químicas, segundo dados da Pesquisa Nacional de Saúde, Brasil, 2013.

	OR (IC 95%)			
	Modelo bloco 1 – variáveis sociodemográficas	Modelo bloco 2 – hábitos de vida	Modelo bloco 3 – situação de saúde	Modelo final
Sexo				
Masculino	1,00			1,00
Feminino	0,75 (0,68–0,84)			0,74 (0,66–0,82)
Faixa etária (anos)				
18–24	1,00			1,00
25–54	1,28 (1,09–1,50)			1,26 (1,07–1,48)
≥ 55	0,92 (0,75–1,13)			0,92 (0,75–1,13)
Nível de instrução				
Superior	1,00			1,00
Médio	1,63 (1,38–1,92)			1,62 (1,37–1,91)
Fundamental ou sem instrução	1,86 (1,58–2,18)			1,77 (1,50–2,08)
Fumante atual				
Não		1,00		1,00
Sim		1,37 (1,22–1,55)		1,21 (1,07–1,37)
Problema de cansaço				
Não			1,00	1,00
Sim			1,28 (1,16–1,43)	1,35 (1,21–1,50)
Dificuldade auditiva				
Não			1,00	1,00
Sim			1,29 (1,08–1,53)	1,24 (1,04–1,48)
Acidente de trabalho				
Não			1,00	1,00
Sim			2,22 (1,75–2,82)	2,00 (1,57–2,54)

OR: *odds ratio*; IC95%: intervalo de confiança de 95%

Nota: Valor de p do ajuste do modelo final = 0,336

Segundo análise multivariada, mulheres (OR = 0,74; IC95% 0,66–0,82) têm menor chance de exposição a substâncias químicas que os homens. Maior chance dessa exposição foi observada no grupo com idade entre 25 e 54 anos (OR = 1,26; IC95% 1,07–1,48) em relação àqueles com idade de até 25 anos, e no que informou nível fundamental de instrução ou sem instrução (OR = 1,77; IC95% 1,50–2,08) em relação àqueles com nível superior de instrução. Indivíduos com nível de instrução médio (OR = 1,62; IC95% 1,37–1,91) também tiveram maior chance de exposição a substâncias químicas quando comparados àqueles de nível superior. Indivíduos fumantes atuais (OR = 1,21; IC95% 1,07–1,37), com problemas de cansaço (OR = 1,35; IC95% 1,21–1,50), com dificuldade auditiva (OR = 1,24; IC95% 1,04–1,48) e que relataram ter sofrido acidente de trabalho (OR = 2,00; IC95% 1,57–2,54) apresentaram maior chance de exposição a substâncias químicas quando comparados aos de condição antagônica (Tabela 3).

Considerando o modelo final de regressão logística binária, foram estimadas as prevalências de exposição a substâncias químicas, com respeito aos perfis ilustrados na Figura 2. A probabilidade de exposição a substâncias químicas foi de 13,8% entre mulheres com idade entre 18 e 24 anos, com nível superior de instrução, que não fumam atualmente, que não relataram problemas de cansaço ou dificuldade auditiva e não sofreram acidente de trabalho nos últimos 12 meses. Por outro lado, observou-se aumentar para 67,1% a probabilidade de exposição no grupo de homens com idade entre 25 e 54 anos, sem instrução ou de nível fundamental, que fumam atualmente e relataram problemas de cansaço ou dificuldade auditiva e que sofreram acidente de trabalho no último ano.

**Figura 2:** Diagrama de probabilidades de exposição a substâncias químicas, segundo modelo final de regressão logística, com dados da Pesquisa Nacional de Saúde (PNS), Brasil, 2013.

## DISCUSSÃO

De acordo com os dados colhidos em domicílio, quase um quinto dos trabalhadores brasileiros, independentemente do vínculo empregatício ou tipo de remuneração, relataram manuseio de substância química no trabalho. Ainda que elevada, a ocorrência está abaixo das estatísticas registradas nos Estados Unidos e na França: 22,3%^20^ e 30%^21^, respectivamente. Quando observados homens e mulheres separadamente, os resultados estão acima do registrado em Honduras e na Argentina, mas abaixo do que foi encontrado no Equador^22^. Associações positivas com perda auditiva, tabagismo e história de acidentes de trabalho, assim como a associação inversa com o nível de escolaridade, além das diferenças de gênero, são consistentes. A ausência de associação com diagnóstico de asma ou bronquite asmática foi, em certa medida, inesperada.

Sobre a maior ocorrência da exposição ocupacional no grupo dos homens, não há surpresas. Há mais homens absorvidos nos setores em que predominam manuseio ou contato com substâncias químicas. As configurações de identidades masculina e feminina se reproduzem na distribuição de homens e mulheres no mercado de trabalho. Os primeiros predominam na indústria em geral, na construção civil e no setor extrativista, setores em que é ampla a produção e utilização de químicos nos processos específicos^23^.

Observaram-se diferenças regionais na exposição a substâncias químicas, indicando associação com a geoeconomia no país. O autorrelato de exposição foi quase o triplo em estados da região Sul quando comparados aos da região Nordeste. Esclarecimentos sobre a heterogeneidade regional da indústria brasileira são úteis para interpretar esse resultado. Na década de 1990, inúmeras empresas industriais se deslocaram ou se instalaram na região Sul, que é próxima aos países do Mercosul, provavelmente atraídas pela boa infraestrutura, salários inferiores aos da região Sudeste e atuação reconhecida na guerra fiscal^24^. Quando se analisaram as unidades da federação mais importantes para cada atividade econômica, os autores constataram a pujança de Minas Gerais e São Paulo para a indústria, seguidos, sem distinção entre eles, pelos estados do Ceará e do Rio de Janeiro, além dos três estados do Sul^25^. Tem-se assim uma aproximação com o mapa da exposição a substâncias químicas. Seria desejável continuar estudando os setores da produção com níveis mais finos de desagregação^26^. O novo cenário de produção, consumo e configuração do mercado de trabalho provavelmente traz elementos para interpretar a maior ocorrência da exposição em regiões consideradas menos industrializadas, pois setores de conservação e limpeza, presentes em todas as regiões, além das atividades na agroindústria, incorporam de forma abundante as substâncias químicas.

A associação com o relato de dificuldade auditiva evoca o conhecimento sobre o efeito ototóxico de várias substâncias químicas potencialmente capazes de provocar alterações neurossensoriais no órgão da audição. Dentre os principais agentes químicos que podem levar a alterações auditivas, incluem-se solventes metais e agrotóxicos organofosforados^26^.

Quanto à associação com acidentes de trabalho, está reconhecida a exposição combinada a vários riscos, principalmente na indústria^27^. A maioria das substâncias químicas manuseadas nos processos de trabalho provocam efeitos neurológicos, que diminuem a concentração e a vigília^28^. É plausível supor existir uma relação entre esse efeito e a ocorrência de acidentes. Ademais, ambientes mais poluídos coincidem com aqueles de maior risco para acidentes. Tais constatações justificam o interesse em abordar a contribuição combinada dos riscos ocupacionais^27^.

A incidência de asma em adultos é frequentemente relacionada à exposição ocupacional, sendo, por exemplo, o problema de saúde mais frequente entre os trabalhadores da indústria moveleira na Turquia^29^. A asma é considerada ocupacional quando se identifica uma relação entre os sintomas e a substâncias presentes no ambiente laboral. Os episódios são induzidos por sensibilização a uma substância específica inalada pelo trabalhador. Outro quadro frequente diz respeito ao agravamento da asma pré-existente por um estímulo presente no local de trabalho^30^. Sobre a ausência de associação observada na amostra da PNS, provavelmente se trata de um efeito do viés de seleção^31^, pois as pessoas intervêm em suas próprias situações, por exemplo, mudando de emprego ou se desvencilhando, quando possível, do manuseio de produtos químicos para evitar o desencadeamento dos sintomas respiratórios. Limites relacionados à cobertura dos sistemas de saúde são possíveis, pois a pergunta do questionário evocava diagnóstico médico.

A associação positiva com cansaço era esperada. Pintores, envernizadores, frentistas e agricultores estão frequentemente expostos aos solventes orgânicos que compõem os produtos utilizados no desenvolvimento de suas tarefas. Muitos sintomas relacionados a essas substâncias são inespecíficos, incluindo náuseas, dificuldade de concentração e atenção, distúrbios do sono, fadiga e cansaço anormal^28^. Na Turquia, 39,8% dos trabalhadores da indústria moveleira, cujos ambientes são geralmente contaminados pelos subprodutos das tintas e vernizes, se queixaram de cansaço anormal^29^. Nas plantações de café da República Dominicana, 76% dos trabalhadores expostos a várias classes de pesticidas relataram cansaço anormal, em contraposição a 39% dos não expostos^4^.

Geralmente, trabalhadores menos escolarizados ocupam postos de trabalho mais perigosos. Tem-se assim a lei inversa do risco: a sobreposição de fatores nocivos tende a ser inversamente proporcional ao estoque de recursos e grau de poder dos indivíduos e grupos atingidos para modificar a situação em que se encontram^20^. Entre os fatores agravantes dos efeitos da exposição a substâncias químicas, estão a falta de informação sobre segurança, equívocos nas operações de pulverização e fracos dispositivos de proteção pessoal, que têm relação com a baixa escolaridade dos trabalhadores^6^. Além disso, a baixa escolaridade está relacionada a menos recursos para elaboração de enfrentamentos positivos, de onde se obtém elementos para interpretar a adoção de hábitos menos saudáveis – no caso, o tabagismo.

Associações significativas entre hábito tabagista e exposição ocupacional a substâncias químicas são consistentes e preocupantes. Observou-se anteriormente que, no grupo dos fumantes, são mais elevados os níveis sanguíneos de substâncias geradas no ambiente laboral do que em seus colegas não fumantes^29^. A literatura traz evidências sobre a maior chance de indivíduos expostos a ambientes laborais insalubres informarem consumo de cigarros como um enfrentamento negativo ao estresse originado naquelas situações^32^. Dados da PNS 2013 permitiram aos autores observar a redução da prevalência de fumantes de tabaco no período analisado (2008 a 2013)^33^. Persistem, contudo, disparidades de gênero, haja vista menor redução no grupo dos homens. É plausível supor sobre a influência das condições laborais nesse âmbito, uma vez que postos mais poluídos alocam mais homens do que mulheres^23^.

No campo da higiene ocupacional, publica-se sobre a diminuição da magnitude da exposição a substâncias químicas no local de trabalho. Provavelmente, contribui para essa realidade o avanço dos programas de saúde do trabalhador no controle da exposição, além da redução do efetivo nas indústrias tradicionais e perigosas, como mineração de carvão, fundição de ferro e aço, extrativismo mineral etc. No conjunto, seriam reflexos da mudança na estrutura ocupacional que se expressa em três frentes: diminuição da força de trabalho agrícola; tendência decrescente no nível ocupacional na indústria, na construção e na mineração; e expansão da absorção da mão de obra no setor de serviços^2^. Essa realidade levou a uma oscilação do pêndulo em favor de outras prioridades de saúde ocupacional, como estresse no trabalho, acidentes devido a quedas e problemas musculoesqueléticos. Por outro lado, em setores cuja maior expressão na absorção da força de trabalho se deu recentemente, os trabalhadores se encontram rotineiramente expostos a produtos químicos no decorrer de seu trabalho (por exemplo, indústria do lazer e entretenimento e empresas de conservação e limpeza). Reconhecidamente relevantes, seja pela magnitude, seja pela agressividade dos efeitos dessas substâncias sobre a capacidade laboral, dados recentes sobre carga de doença e fração atribuível aos riscos ocupacionais para o câncer^6^ na população brasileira reforçam a importância de continuar tentando identificar a exposição ocupacional.

No questionário da PNS, uma única pergunta indagava sobre a exposição a substâncias químicas. Ainda que o conteúdo da pergunta coincida com a pergunta-filtro da maioria dos instrumentos testados e utilizados em recentes inquéritos nacionais e transnacionais focados em amostras de trabalhadores^15^, vieses de informação são esperados dos autorrelatos obtidos de entrevistas face a face^10^.

Obteve êxito o esforço recente dos pesquisadores em padronizar questões sobre as condições de trabalho a fim de fomentar a realização periódica de inquéritos nos países latino-americanos, assim como incrementar a possibilidade de comparação^34^. O questionário CTESLAC indica duas questões para o tópico substâncias químicas: Q27. *Manipula, aplica o está en contacto con sustancias químicas nocivas/tóxicas*? e Q28. *Respira sustancias químicas en forma de polvo, humos, aerosoles, vapores, gases y/o niebla* (*excluido el humo de tabaco*)?^15^. Os impasses para criar consensos nos instrumentos de pesquisa estruturam redes internacionais de pesquisadores. Até o momento, a pergunta do questionário da PNS coincide com uma das perguntas do questionário já aplicado nos países latino-americanos e na União Europeia. Espera-se, contudo, esforço para aprimorar o questionário, de maneira a completar as perguntas e favorecer o desejado consenso.

O déficit de informações confiáveis e de qualidade incitam os pesquisadores a buscar junto às populações informações autorreferidas sobre uma ampla variedade de exposições, como uso de medicamentos, dieta, tabagismo e histórico ocupacional. Utilizar autorrelatos para definir expostos e não expostos e para estimar a duração da exposição constitui um dos componentes críticos da maioria dos estudos epidemiológicos. Entretanto, resultados de estudos específicos, como a verificação da qualidade das informações autorreferidas sobre a exposição a agrotóxicos, encorajaram pesquisadores a manter e aplicar questionários por meio de entrevistas^35^.

Ao contrário das informações sobre doenças e acidentes, que também são fundamentais, estudos sobre a exposição têm a vantagem de facilitar a intervenção oportuna e eficaz porque antecedem aos efeitos e prejuízos. Espera-se que os resultados apresentados aqui contribuam para instigar a (re)formulação de perguntas sobre exposição ocupacional em futuros inquéritos nacionais de saúde, pois os vários sistemas de informação existentes estão fragmentados, geralmente incompletos, seja porque não abrangem a população informalmente inserida no mercado de trabalho, seja porque dependem de sistemas ainda não consolidados^6^.

Possivelmente, os resultados da PNS sobre o autorrelato de exposição a substâncias químicas serão informações valiosas para os sistemas de vigilância à saúde dos trabalhadores, além de fornecer pistas para as investigações sobre os fatores de risco para a carga de doenças crônico-degenerativas. Por fim, resultados obtidos reforçam indicações anteriores quanto à pertinência de se levar em conta as características do emprego e do trabalho nos programas visando a cessação do tabagismo na população^32^.

## CONCLUSÕES

A ocorrência de autorrelato de exposição a substâncias químicas no trabalho mostrou-se elevada, além de possivelmente subestimada. Exceto pela ausência de associação com asma ou bronquite asmática, em que problemas de cobertura devem ser considerados, as associações positivas com cansaço, dificuldade auditiva, acidentes de trabalho e tabagismo, bem como a associação inversa com o nível de escolaridade, além das diferenças de gênero, são consistentes. O viés de informação em função do conteúdo e formato da questão que orientou a construção da variável principal impõe limites para comparar os resultados, ainda que esses estejam em convergência com um panorama recentemente descrito nos países da América Latina e Caribe. Com vistas a suprir as lacunas nas investigações sobre doenças crônicas não transmissíveis, sugere-se aperfeiçoar o instrumento de coleta da PNS na dimensão ocupacional.
